# Nonlinear cooling and control of a mechanical quantum harmonic oscillator

**DOI:** 10.1126/sciadv.aee5380

**Published:** 2026-11-11

**Authors:** Matteo Simoni, Ivan Rojkov, Matteo Mazzanti, Wojciech Adamczyk, Alexander Ferk, Pavel Hrmo, Shreyans Jain, Tobias Sägesser, Daniel Kienzler, Jonathan Home

**Affiliations:** ^1^ Institute for Quantum Electronics, ETH Zürich, Otto-Stern-Weg 1, 8093 Zürich, Switzerland.; ^2^ Quantum Center, ETH Zürich, 8093 Zürich, Switzerland.

## Abstract

Nonlinearities are a key feature allowing nonclassical control of quantum harmonic oscillators. However, when nonlinearities are strong, designing protocols for control is often difficult, placing a barrier to exploiting these properties fully. In this work, using a single trapped-ion oscillator operated in the strongly nonlinear regime of the atom-light interaction, we show how to generate localized multi (two, three, four, and five)–component Schrödinger’s cat manifolds using a novel form of nonlinear reservoir engineering. We then specifically select Hamiltonians which allow us to perform measurements on these state manifolds. Our work demonstrates the experimental use of high-order nonlinear processes for control of nonclassical states of a quantum harmonic oscillator, opening up a new toolbox which can be applied to bosonic quantum error correction, computation, and sensing.

## INTRODUCTION

The quantum harmonic oscillator serves as a foundational model for many systems close to equilibrium and as a key tool in the realization of quantum technologies, from error-corrected qubits (*1*–*10*) to precision sensors and transducers (*11*–*19*). These have the potential to be applied to areas as diverse as probing fundamental physics (*20*–*25*) and information technologies (*26*–*30*). The states of an oscillator that exhibit more exotic and interesting quantum mechanical features are non-Gaussian states (*31*). In addition, non-Gaussian resources are required to access the full power of quantum computers based on continuous variable systems (*32*–*34*). Since quantum oscillators follow linear equations of motion, an additional nonlinearity has to be introduced to generate non-Gaussian features (*31*, *35*, *36*). As nonlinearity extends to higher order, a broad range of effects comes into play, opening up a number of opportunities for quantum state control and stabilization including, for instance, the control of states with higher-order rotational symmetry (*28*, *29*, *37*, *38*).

It has been common both in mechanical and electromagnetic field oscillators to gain a source of nonlinearity from a linear coupling to an ancilliary system with anharmonic levels selected through resonance conditions, realizing an effective two-level spin (*39*–*42*). However, achieving higher-order nonlinearity then typically results in parasitic off-resonant couplings which require careful consideration (*43*–*46*). The alternative approach, based on an interaction which has a nonlinear form, is relatively unexplored. Atomic systems offer a spin-oscillator coupling Hamiltonian which is inherently nonlinear, due to the sinusoidal form of the laser or microwave fields (*47*). This nonlinear nature was considered in a number of early theoretical works (*48*–*51*) as well as for the generation of large amplitude oscillator states (*52*–*54*). However, for much work on quantum oscillator control, the interaction is linearized by working in the so-called Lamb-Dicke regime, in which the wavelength of the light is much longer than the oscillator wave packet (*55*), such that the spin itself is the source of nonlinearity. In this context, the non–Lamb-Dicke behavior has primarily been considered as a limitation to control (*56*, *57*). The simple analytical expressions of the Lamb-Dicke regime have facilitated the design of protocols used for laser cooling (*58*), state engineering (*59*), and multi-qubit quantum gates (*60*) as well as bosonic error correction using codes constructed from translation symmetry (*7*). However, in this linearized regime, the coupling to higher-order nonlinearities is by definition weak (*40*). The experimental realization of high-order nonlinearities in the regime where quantum effects are controllable remains challenging across a range of different platforms (*61*–*64*).

In this work, we make full use of the nonlinear nature of the atom-light coupling by operating our trapped-ion setup outside the linearized Lamb-Dicke regime, using it to produce and control stabilized manifolds of quantum states. This introduces a resource of nonlinearity distinct from the spin, enabling the realization of high-order boson processes which can be selected through resonance conditions. We first show how this allows to implement nonlinear reservoir engineering (NLRE) (*65*) which we show prepares manifolds of Schrödinger’s cats with two-, three-, four-, and fivefold rotational symmetries inherited from the drive Hamiltonian. This allows us to demonstrate experimentally the stabilization of manifolds of three-, four-, and five-component cat states in a mechanical system. We also demonstrate control of these states by applying Hamiltonians composed of a single high-order resonant process with an approximately linear matrix element dependence over the limited range of occupied states, allowing the mapping of the symmetry of the underlying state onto a spin readout. We use this to demonstrate an *n* mod 3 measurement which allows us to purify mixtures of three component manifolds. Our work shows that harnessing the native nonlinearity of the atom-light coupling allows us to implement high-order dissipative and unitary dynamics. This provides a tool for accessing a broad range of nonlinear physics, including potentially for realizing high-order rotationally symmetric bosonic error-correction codes for quantum computing.

## RESULTS

### Trapped-ion physics outside Lamb-Dicke regime

In the experiments described below, the mechanical oscillator in question is the center of mass motion of the ion in the trap, which oscillates with a frequency $\omega_{o}$. This is coupled to two internal electronic states of the ion (separated in energy by $\hbar \omega_{s}$) using a traveling wave light field with wave vector *k* and frequency $\omega_{L}$. The coupling Hamiltonian can be written as $\hat{H}=\hbar g{\hat{\sigma }}_{+}e^{\mathrm{ik}\hat{x}-i\left(\omega_{L}-\omega_{S}\right)t}+H.c.$, which is a nonlinear function of the position $\hat{x}$. *g* quantifies the coupling of the laser field to the internal-state transition. Since the position $\hat{x}$ oscillates, the coupling of the light to the internal states is phase modulated, producing modulation sidebands which can be selectively driven by setting the laser to frequencies $\omega_{L}=\omega_{s}+\Delta n\;\omega_{o}$ with Δn an integer. Atomic experiments have, for many decades, mostly worked in the regime where $|\langle k\hat{x}\rangle |\ll 1$, known as the Lamb-Dicke regime, for which the lowest sidebands dominate and a first-order expansion of the $e^{\mathrm{ik}\hat{x}}$ term linearizes the coupling. Outside of this linearized regime, the matrix elements for resonant sidebands of $\hat{H}$ coupling Fock states ∣n〉 and ∣n+Δn〉 are approximately given by Bessel functions following

$$\langle ↓|\langle n|\hat{H}|n+\Delta n\rangle |↑\rangle ≃\hbar g\;J_{\Delta n}\left(2\eta \sqrt{n+\frac{\Delta n+1}{2}}\right)$$ (1)

with $\eta ={\mathrm{kx}}_{0}$ the Lamb-Dicke parameter and $x_{0}=\sqrt{\hbar /\left(2m\omega_{o}\right)}$ the zero-point extent of the ground state of the oscillator. These matrix elements are more commonly expressed in terms of Laguerre polynomials and factorials. The approximate expression in Eq. 1 is numerically and analytically more convenient and is accurate to within 1% (*65*, *66*).

Figure 1 compares the coupling strength of boson processes up to fourth order. Inside the Lamb-Dicke regime, the light field can be approximated as linear across the oscillator wave function (1a), rendering processes of order Δn>1 weak (1c). This is not the case outside the Lamb-Dicke regime (1b), where high-order processes have nonnegligible coupling (1d), an effect which we use explicitly in the work below.

**Fig. 1.**
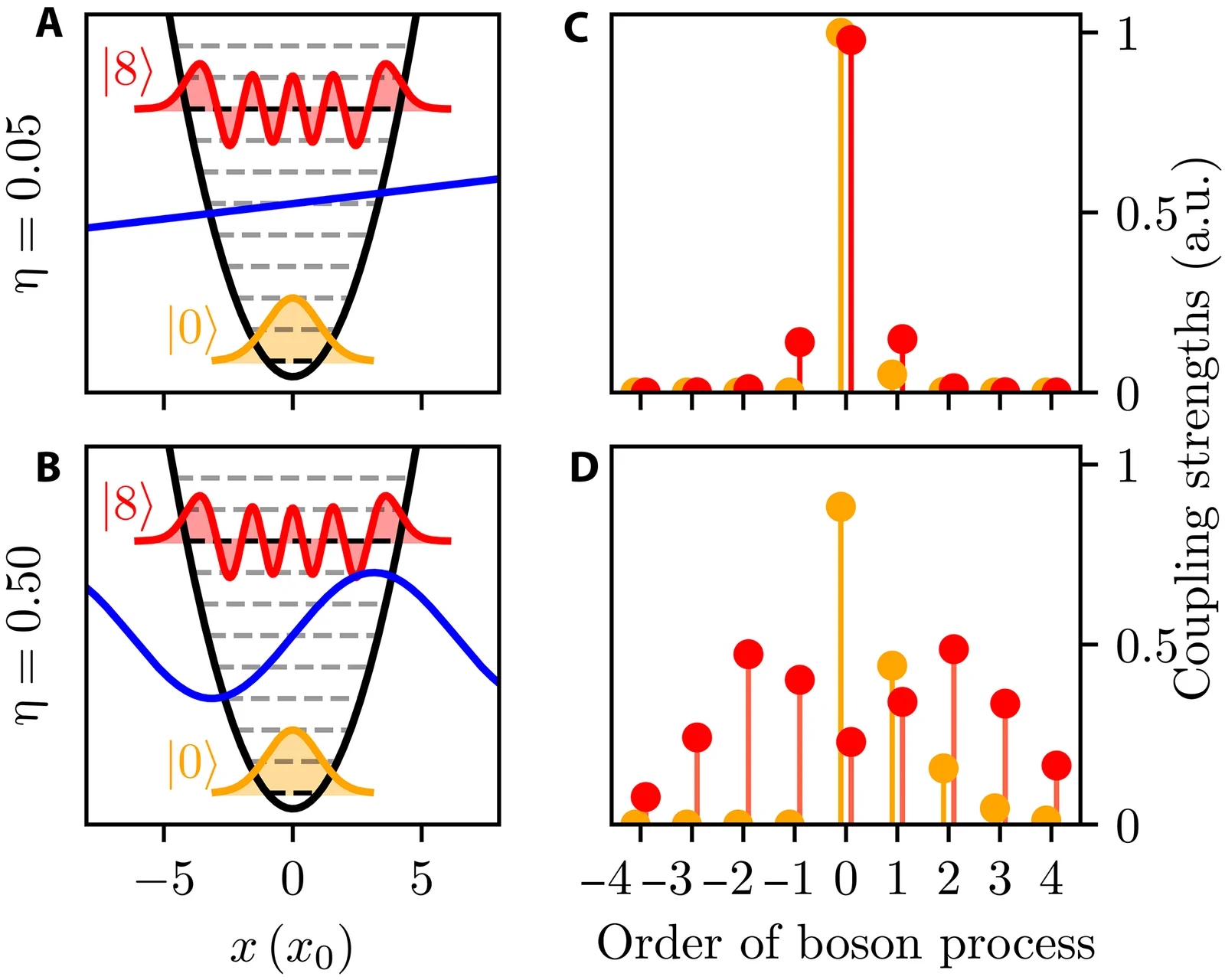
Nonlinear light matter interaction. Pictorial representation of inside (**A**, η=0.05) and outside (**B**, η=0.50) Lamb-Dicke regime physics. The black parabolas represent the harmonic oscillator with Fock states ∣0〉 (in orange) and ∣8〉 (in red). The blue solid line is the incoming light field. The Lamb-Dicke parameter η is determined by the wavelength of the laser. The coupling strength of boson processes from order −4 to +4 is presented inside (**C**) and outside (**D**) the Lamb-Dicke regime, showing that high-order nonlinear processes can be strongly driven outside the linearized regime.

### Nonlinear reservoir engineering

We first demonstrate cooling of the motional mode into manifolds of non-Gaussian states by NLRE (*65*), performed by simultaneously driving an excitation raising ($\Delta n_{1}>0$, $r=\Delta n_{1}$) and an excitation lowering ($\Delta n_{2}<0,l=-\Delta n_{2}$) resonant sideband while also continuously optically pumping the spin at a rate γ. Under conditions for which the sideband drives are weaker than γ, the spin can be adiabatically eliminated, resulting in a master equation $\dot{\hat{\rho }}=\hat{L}\hat{\rho }{\hat{L}}^{†}-\left({\hat{L}}^{†}\hat{L}\hat{\rho }+\hat{\rho }{\hat{L}}^{†}\hat{L}\right)/2$ for the motion alone (*67*) with the Lindblad jump operator

$$\hat{L}\propto \sum_{n}|n\rangle \left[\langle n-r|-\frac{\Omega_{l}\left(n\right)}{\Omega_{r}\left(n-r\right)}\langle n+l|\right]$$ (2)

which combines an excitation raising process of order *r* with strength $\Omega_{r}\left(n\right)=g_{r}J_{r}\left(\eta ,n,r\right)$ and an excitation lowering process of order *l* with strength $\Omega_{l}\left(n\right)=g_{l}J_{l}\left(\eta ,n,l\right)$. Since the strength of these functions varies with *n*, crossing points occur where $\Omega_{r}\left(n^{*}\right)=\Omega_{l}\left(n^{*}\right)$. If $\Omega_{l}\left(n\right)<\Omega_{r}\left(n\right)$ for $n<n^{*}$ and $\Omega_{l}\left(n\right)>\Omega_{r}\left(n\right)$ for $n>n^{*}$, then for each experimental run, population accumulates around the crossing point in a pure dark state $|\psi_{m}\rangle$ for which $\hat{L}|\psi_{m}\rangle =0$. Across different runs, the steady-state manifold consists of a statistical mixture of the various $|\psi_{m}\rangle$. Since the operator in Eq. 2 only involves the terms ∣n〉〈n−r∣ and ∣n〉〈n+l∣, each dark state is a superposition of Fock states that are d=r+l apart. Therefore, we write these in the form $|\psi_{m}\rangle =\sum_{k}c_{k}|m+d\;k\rangle$, with $c_{k}$ such that the 〈n−r∣ and 〈n+l∣ terms in $\hat{L}$ destructively interfere. This periodic *d*-fold superposition of Fock states corresponds to a *d*-dimensional rotational symmetry in the $\hat{x}-\hat{p}$ phase space (*37*). This can be seen by applying the rotation operator $\hat{R}\left(2\pi /d\right)=\text{exp}\left\{i\left(2\pi /d\right)\hat{n}\right\}$ to $|\psi_{m}\rangle$, for which we find $\hat{R}\left(2\pi /d\right)|\psi_{m}\rangle =\text{exp}\left\{i\left(2\mathrm{\pi m}/d\right)\right\}|\psi_{m}\rangle$, i.e., $|\psi_{m}\rangle$ is an eigenstate of the rotation operator. Given that *m* is only defined modulo *d*, $|\psi_{m}\rangle =|\psi_{m+d}\rangle$ and a manifold of states spanned by *d* different dark states is stabilized. Since the $|\psi_{m}\rangle$ have the same energy, also their superpositions are stabilized (*65*).

We engineer NLRE dissipative operators composed of processes of orders (r,l)=(0,2),(1,2),(1,3),(2,3) and examine their steady state. Experiments use a single beryllium ion confined in the microfabricated surface Penning trap presented in (*68*). The quantized mechanical oscillator is the motion along the magnetic field axis, with a typical oscillator frequency $\omega_{o}=2\pi \times 2.0$ MHz. The spin degree of freedom is encoded in two energy levels of the $S_{1/2}$ manifold which we denote ∣↑〉 and ∣↓〉 following the convention in (*68*). The spin-oscillator coupling is mediated by the light field of a pair of orthogonal Raman beams at 313 nm resulting in η∼0.5. Optical pumping from ∣↓〉 to ∣↑〉 is performed via a short-lived internal state. The experimental sequence starts by cooling the ion close to its motional ground state [thermal distribution with $\bar{n}=0.007\left(3\right)$] using Doppler and sideband cooling (*68*), with the NLRE subsequently applied. To implement the NLRE dissipative operator, we use the Raman beams to drive a bichromatic pulse composed of two laser tones at frequencies $\omega_{L}=\omega_{s}+r\omega_{o}$ and $\omega_{L}=\omega_{s}-l\omega_{o}$ while simultaneously performing optical pumping. The relative phase between the two tones determines the orientation of the manifold in phase space. A miscalibration of $\omega_{o}$ results in an undesired time-dependent drift of this phase over the stabilization time. More details on the calibrations of the NLRE operator can be found in Materials and Methods. A variation to this sequence was implemented for (r,l)=(0,2), where after ground-state cooling, the oscillator was prepared in a two-headed cat state close to the dark state before applying NLRE.

We drive the NLRE for a time sufficient to attain the steady-state mixtures of $|\psi_{m}\rangle$ with $\hat{L}|\psi_{m}\rangle =0$, which is found by monitoring of the spin population (*69*). To diagnose the resulting states, we reconstruct the Fock probability distribution using standard techniques (*59*), as well as the Wigner functions from measurements of the real part of the characteristic function (*70*) using maximum likelihood estimation (MLE) (more details can be found in Materials and Methods). The results are shown in Fig. 2. We overlay the Fock populations with the magnitude of the relevant matrix elements for the raising and lowering processes. The results show clearly that population accumulates around the crossing points of the latter. The Wigner functions show the predicted *d*-fold rotational symmetry, which is also visible through the *d*-fold periodicity in the Fock state distributions. In both the Fock state populations and the marginals of the Wigner functions, we also overlay numerical simulations which include the Hamiltonian and optical pumping, but not experimental imperfections such as photon recoil, motional dephasing and heating, and imperfect ground-state cooling. Nevertheless we see good agreement with the experimental results, which illustrates the robustness of the reservoir engineering technique. In particular, as long as a stable crossing point is present, a dark manifold will be stabilized, with fluctuations of experimental parameters only slightly changing the shape of the steady states. Operating in the regimes $g_{l}/g_{r}\ll 1$ or η≫1 (not explored in this work) would require considering the presence of multiple crossing points, which could result in population leakage to other dark manifolds at larger Fock numbers, allowing for the dissipative stabilization of multiple dark manifolds at once.

**Fig. 2.**
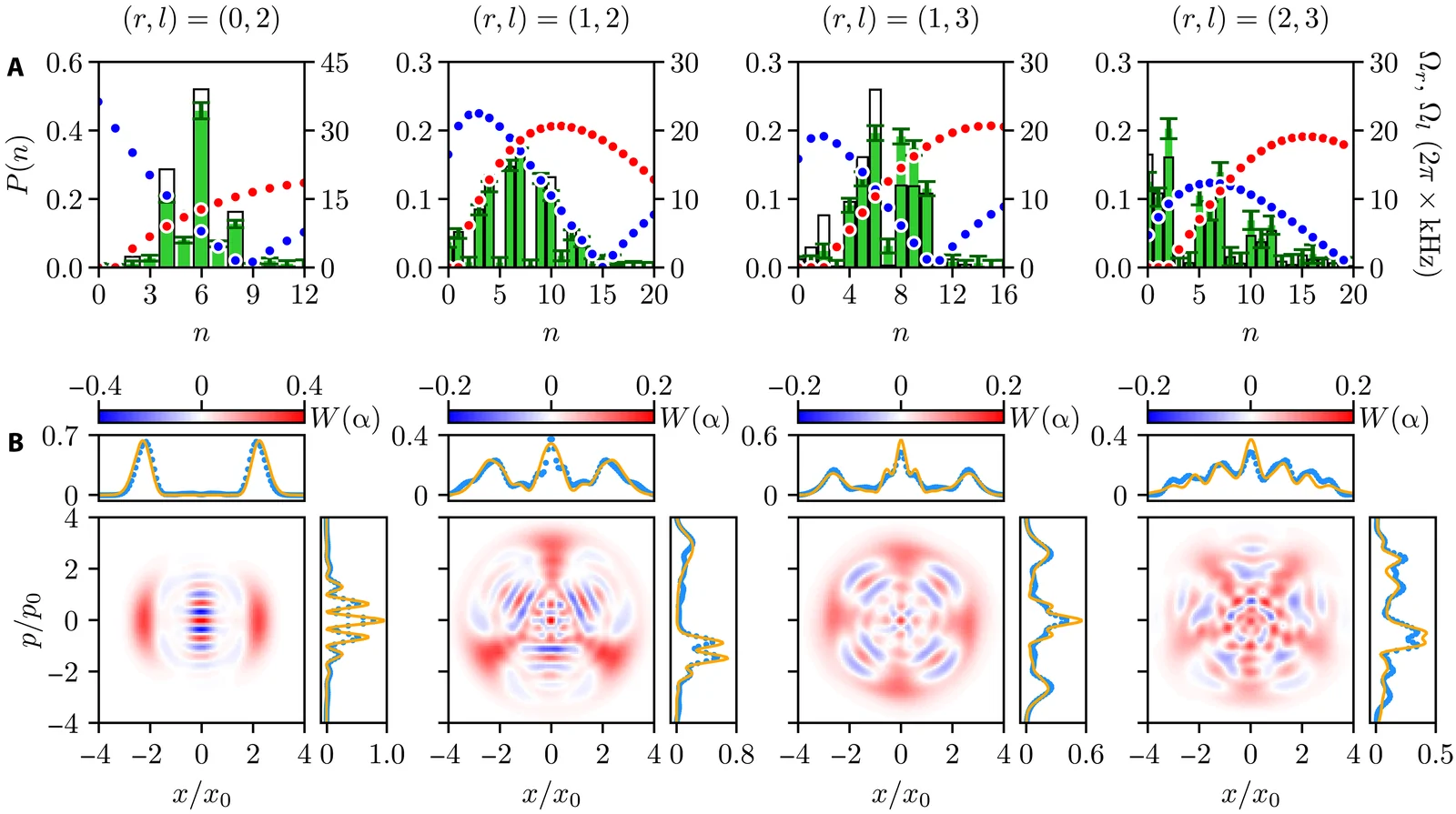
NLRE with boson processes of orders (r,l)=(0,2), (1,2), (1,3), (2,3). (**A**) Steady-state Fock state distributions. Experimental results (green bars) include error bars given as SEM. Results of simulations are overlain (black outlines). Also shown are the matrix elements of the raising (blue) and lowering (red) processes. (**B**) Steady-state Wigner functions, reconstructed using MLE. The marginals, defined as $\int_{-\infty }^{\infty }dp\;W\left(x,p\right)$ and $\int_{-\infty }^{\infty }dx\;W\left(x,p\right)$ for *x* and *p*, respectively, are shown at the sides (blue points), along with results of numerical simulations which do not include experimental imperfections (orange lines). Error bars on the marginals are not included due to the errors being correlated; the full covariance matrices of each reconstructed density matrix are shown in fig. S4.

We compute the fidelities between the reconstructed density matrices and the simulated ones and obtain 89.7(4)%, 86(1)%, 87(1)%, and 82(2)% for (r,l)=(0,2),(1,2),(1,3),(2,3) respectively, with the uncertainty obtained through bootstrapping (see Materials and Methods). The negativity of the reconstructed Wigner functions demonstrates that NLRE generates manifolds of non-Gaussian states.

We observe that for (r,l)=(1,2),(1,3),(2,3), the steady-state distribution occupies a subset consisting of *l* (2, 3, and 3, respectively) of the total of *d* (3, 4, and 5, respectively) dark states which might be expected from the symmetry of the operator in Eq. 2 (*65*). This is the result of a leakage process happening close to the ground state due to a lack of interference. For m≥l, the lowering process from Fock state ∣m〉 to ∣m−l〉 can be driven. However, for the lowest *r* values of *m* for which this happens, there is no boson raising process to provide destructive interference because Fock state ∣n−r〉 in Eq. 2 does not exist. This phenomenon gives direct evidence of the presence of destructive interference in the NLRE. More details on the timescales of the stabilization and leakage can be found in Supplementary Text.

### Amplitude and squeezing of the steady states

The dependence of the boson raising and lowering processes on *n* determines both the crossing point and the rate at which the raising and lowering processes diverge away from this point. The crossing point determines the mean Fock excitation $\bar{n}$, while the divergence determines the variance Var(n). We can modify the crossing point by changing the experimental parameters η and $g_{l}/g_{r}$. Figure 3 shows measurements of $\bar{n}$ and the Mandel Q parameter $Q=\text{Var}\left(n\right)/\bar{n}-1$ (*71*) for five different choices of parameters and with (r,l)=(1,2). These show the ability to vary $\bar{n}$ from 5.1(3) to 10.8(5) and Q from 0.1(2) to 1.1(3) independently. For each measured distribution, we present a simulation of the corresponding Wigner function for illustration. This capability to control the squeezing of the resulting steady states has been proposed for quantum error correction with cat codes, where the number squeezing has been shown to reduce logical error rates (*65*, *72*, *73*).

**Fig. 3.**
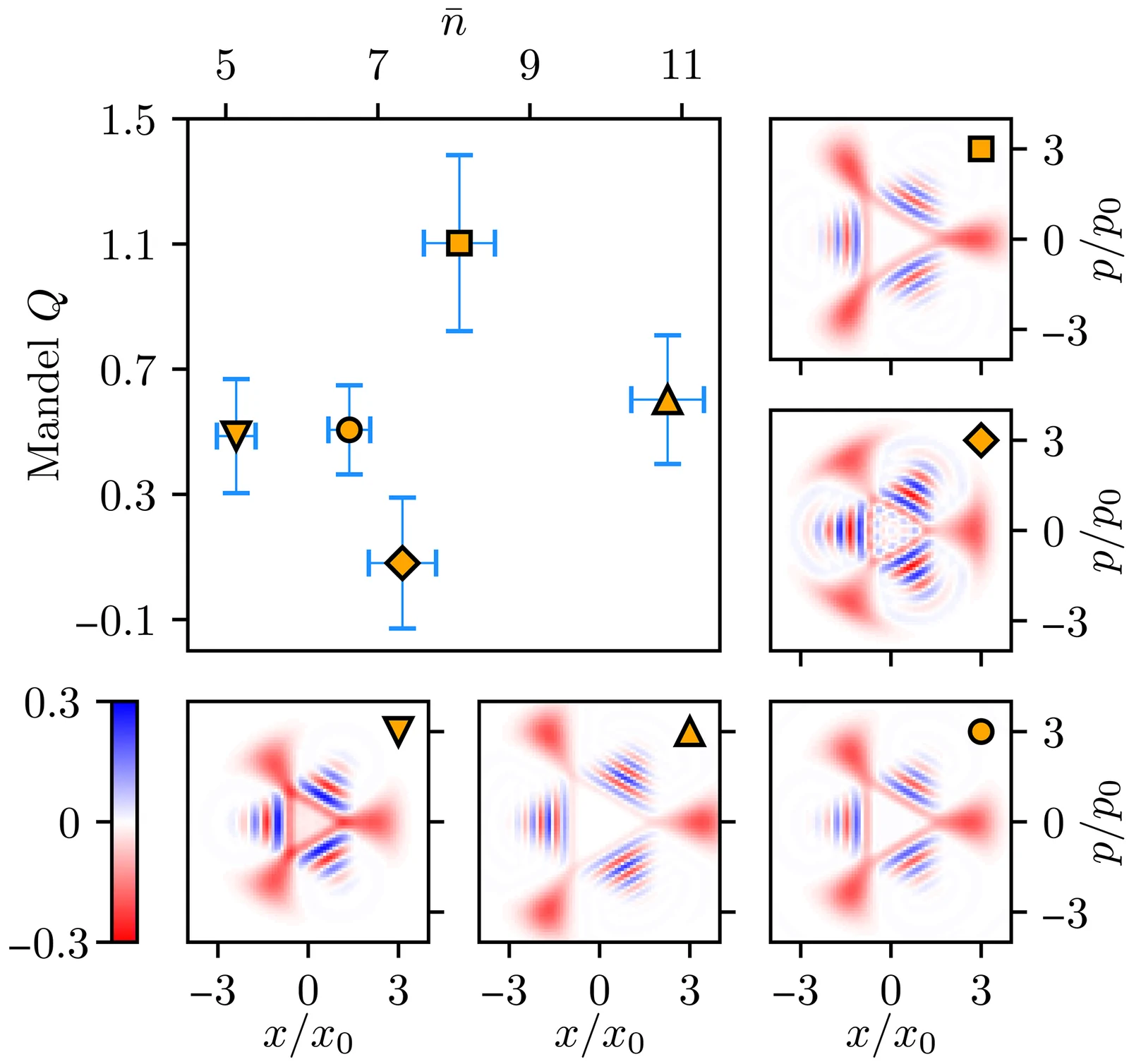
Amplitude and squeezing of the steady state. Mean excitation number $\bar{n}$ and Mandel *Q* parameter extracted from the steady-state Fock distributions of (r,l)=(1,2) when changing the properties of the crossing point. The error bars represent 1 SD. To the right and on the bottom, corresponding simulations of the Wigner functions are shown.

### Nonlinear control Hamiltonians

The presence of nonlinear control Hamiltonians poses the question of how to design techniques for manipulating the oscillator state. Since the states created by NLRE are localized in energy, one option is to choose a particular sideband such that the matrix elements vary in a useful way over the extent of the occupied states. Here, we give a concrete example of Hamiltonian control by initializing one of the two states $|\psi_{0}\rangle$ and $|\psi_{1}\rangle$ stabilized by the NLRE operator (r,l)=(1,2). Our approach relies on the fact that under a Hamiltonian ${\hat{H}}_{\text{lin}}$ whose matrix elements vary linearly with Fock number *n*, each Fock state accumulates a Rabi phase proportional to *n*. Since $|\psi_{0}\rangle$ and $|\psi_{1}\rangle$ occupy Fock states spaced by a constant d=3, there exists a time $t_{\text{rev}}$ after which the states $|\psi_{0}\rangle$ and $|\psi_{1}\rangle$ are highly correlated with the internal spin state. For η∼0.5, an appropriate choice is the fourth-order sideband (Δn=4), whose matrix elements, as illustrated in Fig. 4A, are approximately linear across the (r,l)=(1,2) steady-state manifold. We therefore apply this Hamiltonian for the time $t_{\text{rev}}$, measure the spin state through fluorescence detection, and postselect on the outcome (more on this can be found in Supplementary Text). The selection of $t_{\text{rev}}$ is performed by inspecting the numerical simulations of the dynamical evolution shown in Fig. 4B. These show periodic high correlation which occurs at similar (but not identical) times for the two states. For experimental simplicity, we operate with a fixed $t_{\text{rev}}=170\;\mu s$, which produces $P\left(\psi_{1}|↑\right)=P\left(\psi_{0}^{\prime }|↑\right)=0.87$. The prime in the latter indicates that although a single eigenstate would be selected by the measurement, the resulting state is shifted upward by four phonons due to the use of the fourth-order sideband.

**Fig. 4.**
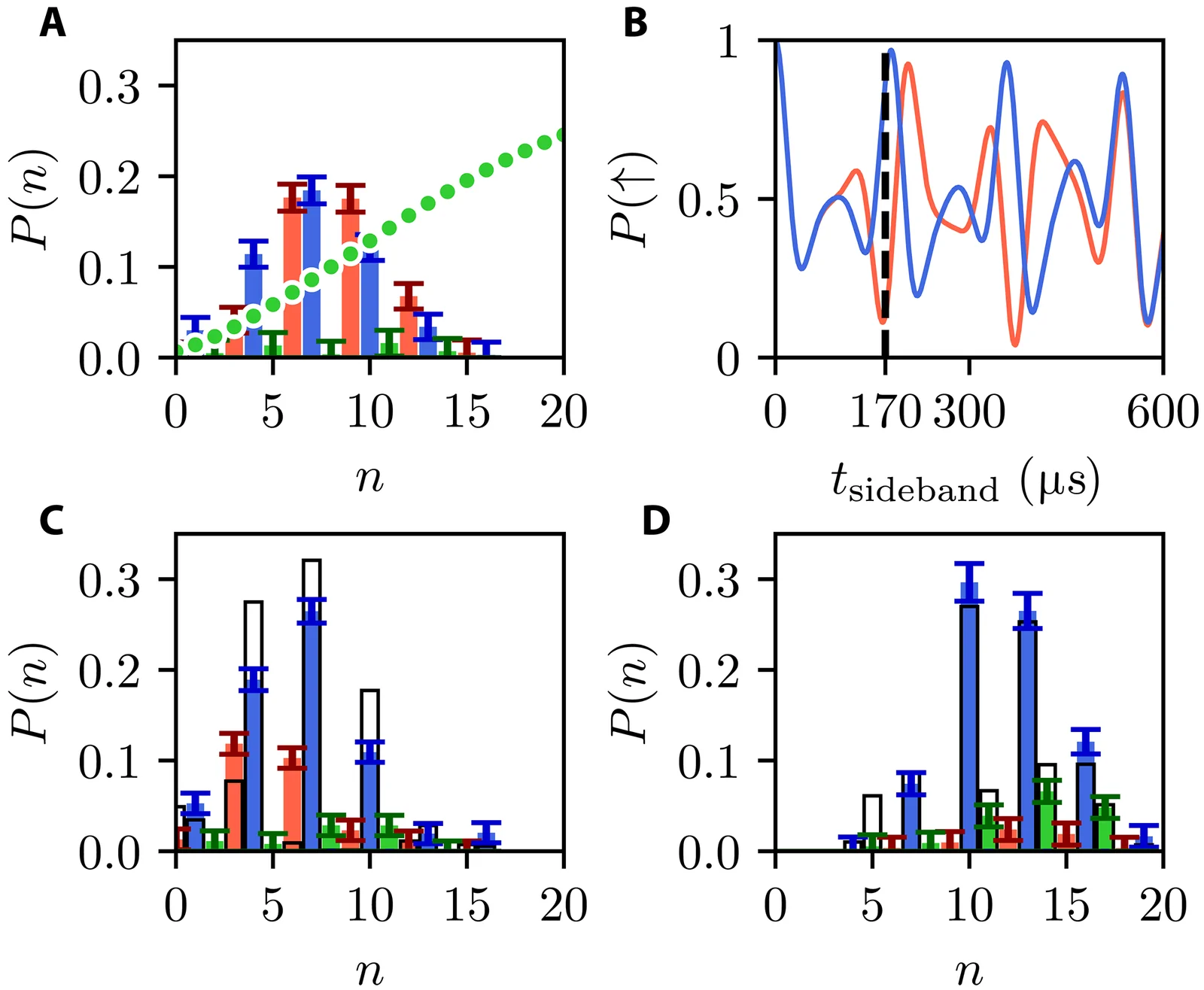
Nonlinear Hamiltonian control. (**A**) Fock distribution of the manifold of states $\left\{|\psi_{0}\rangle ,|\psi_{1}\rangle \right\}$ (in red and blue, respectively) obtained as the steady state of the NLRE operator (r,l)=(1,2). Green dots indicate a simulation of the matrix elements of the fourth-order sideband, showing an approximately linear scaling across the excited Fock states. (**B**) Simulated Rabi flops as a function of the duration of the fourth-order sideband $t_{\text{sideband}}$, starting from $|\psi_{0}\rangle$ (in red) and from $|\psi_{1}\rangle$ (in blue). The vertical dashed black line indicates $t_{\text{rev}}=170\;\mathrm{\mu s}$. We present the measured Fock distributions of $|\psi_{1}\rangle$ (**C**) and of $|\psi_{0}\rangle$ (**D**) extracted from the spin excitation probability as a function of the duration of a fourth-order sideband probe. The error bars are propagated from the fits and correspond to the SEM. Black rectangles show the Fock distributions obtained simulating the postselection sequence and taking into account no experimental imperfection.

For the postselection, we require that the detection does not scatter photons and thus that the ion is in ∣↓〉 during detection. For this reason, we modify the sequence when we want to postselect $|\psi_{1}\rangle$ by performing a spin inversion before detection. Figure 4 (C and D) shows the experimentally measured Fock distributions $P_{i}^{E}\left(n\right)$ of the postselected states, where i=1 if the spin-inversion pulse was used and i=0 if it was not. When we include the spin-inversion pulse, we measure that 66(3)% of the population of the postselected state occupies Fock states corresponding to $|\psi_{1}\rangle$, while for postselection without the spin inversion, we find that the corresponding probability is 77(4)%, considerably higher than the initial value of 47(4)%. These experimental data are accompanied by probability distributions $P_{i}^{S}\left(n\right)$ obtained from simulations of the postselection sequence. The fidelity between the measured and simulated Fock probability distributions $F_{i}={\left[\sum_{n}\sqrt{P_{i}^{E}\left(n\right)P_{i}^{S}\left(n\right)}\right]}^{2}$ gives $F_{0}=0.84\left(9\right)$ and $F_{1}=0.9\left(1\right)$. Further purification of the states could be achieved by using longer sideband probe times which have higher correlation or by repeating the cycle of probing and measurement. This would require better spin (∼1 ms) and oscillator (∼25 ms) coherence than we have presently.

## DISCUSSION

Our work demonstrates the ability to use nonlinear interference to localize state manifolds with rotational symmetry in phase. This ability is at the heart of a number of proposed methods for bosonic error correction, extending beyond the two-component cat codes which have been studied extensively in superconducting circuits (*1*, *28*, *74*) to higher-order rotational symmetries with a potential for stronger protection from errors (*37*). The general approach of NLRE demonstrated here has no strict reliance on the form of the nonlinearity; hence, it has promise for being applied to other nonlinear systems (*65*). For atomic systems, the use of nonlinearity through working outside the Lamb-Dicke regime as demonstrated here paves the way to realizing multicomponent cat codes, which are promising candidates to correct common errors such as oscillator dephasing (*28*) but have thus far not been realized. A change of focus to use non–Lamb-Dicke terms as coherent Hamiltonians offers previously unidentified forms of control, for instance, generalizing Kerr-type Hamiltonians through the use of nonlinearity. One possible approach would be to use the nonlinearity of the carrier Δn=0 term, for which the Bessel function expands as even powers of *n* (*75*). Moreover, hybrid Hamiltonian-dissipative stabilization schemes such as (*76*) promise to be readily implementable and could offer enhanced resilience to the photon recoil intrinsic of dissipative schemes.

Operation in the nonlinear regime poses a range of challenges in terms of control, likely requiring heuristic or numerically optimized methods for designing protocols due to the departure from analytic simplicity (*77*, *78*). Our paper demonstrates two simple approaches to constructing such control, but the development of a more comprehensive range of design tools remains an active area of study. Another open question relates to generalizing these methods to control multiple interacting quantum oscillators. In the trapped-ion approach used here, this could be realized by combining NLRE or related techniques with ions coupled via the Coulomb interaction (*68*) or by using multimode couplings (*79*, *80*). One potential challenge, so far unaddressed, would be the interplay of ion transport and potential reconfiguration with manifolds of stabilized cat states. Another interesting addition would be the generalization of the NLRE approach to systems comprising multiple harmonic oscillators. Overall, this would provide a toolbox for studying coupled driven nonlinear systems, a widely applicable area of active study in classical physics, into the quantum regime (*81*–*85*).

## MATERIALS AND METHODS

### Oscillator frequency calibration

If the oscillator frequency $\omega_{o}$ is miscalibrated, then the frequencies of the two processes of the NLRE operator do not add up to $d\;\omega_{o}$, resulting in a drift of the steady-state manifold in the oscillator frame. A miscalibration of $\omega_{o}$ by only 10 Hz would result, after the 7-ms typical driving time of the NLRE, in a spurious phase of ∼π/7. To calibrate this frequency, we start by ground-state cooling and then apply a short and weak linear electric field that drives the ion to a small displaced state (tickle-out pulse). After a wait time during which the ion oscillates freely, we apply a second identical linear electric field with opposite phase with respect to the first one (tickle-in). We then drive a sideband probe sensitive to fractions of the Fock population outside of the ground state and detect the ion’s fluorescence. If $\omega_{o}$ is properly calibrated, then the tickle-in pulse exactly reverses the displaced state generated with the tickle-out pulse, resulting in no fraction of the Fock population being outside of the ground state. By scanning $\omega_{o}$ and observing the fluorescence, we measure Ramsey fringes whose widths and distance depend on the selected wait time. This procedure allows us to calibrate $\omega_{o}$ with an accuracy of a few hertz. We observe that during experiments that use the sideband cooling lasers, $\omega_{o}$ drifts by about −24 Hz/hour, followed by a slow relaxation of about +2 Hz/hour when the lasers are off. We attribute this to slow charging of the trap electrodes due to the ultraviolet (UV) photons from the lasers. We compensate for this drift by dividing long experimental runs into batches lasting only up to 5 min each, interleaved by a recalibration of $\omega_{o}$. This procedure reduces the drift during each batch to less than −2 Hz, which corresponds to a phase of only ∼π/36 over 7 ms. For the purpose of the experiments carried out in this work, this drift did not limit the speed of operations; therefore, a more detailed study of its dependence of the power of the UV beams was not performed.

### NLRE operator calibration

We find the Lamb-Dicke parameter using $\eta =k\sqrt{\hbar /\left(2m\omega_{0}\right)}$ where $\omega_{o}$ is calibrated as described above and $k\approx 2\pi \sqrt{2}/\left(313\;\text{nm}\right)$ for our pair of orthogonal Raman beams. We calibrate the relative coupling strength of the boson adding and boson removing process by measuring the optical power of each resonant sideband with a photodiode. We estimate the relative uncertainty on the coupling strengths calibrated with this approach to be less than 5%. We calibrate the bare qubit transition frequency $\omega_{s}$ with an accuracy of about 100 Hz using a weak millimeter wave. On top of this, a Stark shift due to the Raman beams common to the two processes constituting the NLRE operator needs to be taken into account. We calibrate it to an accuracy of about 2 kHz (less than 3% of the full width at half maximum) by scanning a common frequency shift while driving the NLRE operator for 500 μs and looking for a dip in the fluorescence signal, indicative of being on resonance.

### Quantum state tomography

The characteristic function of a trapped-ion oscillator can be reconstructed by performing state-dependent displacements (SDD) and by measuring the spin-excitation probability (*70*). Since we operate outside the Lamb-Dicke regime, the SDD is nonlinear, meaning that the resulting nonlinear characteristic function cannot be directly mapped to the Wigner function through a discrete Fourier transform. For this reason, we instead adopt MLE to recover the density matrix of the state and consequently the Wigner function (*86*). We choose α such that it parametrizes a M×M grid of points in phase space. For each α, we acquire statistics by performing *N* independent projective measurements of ∣↑〉 or ∣↓〉 through fluorescence detection. The likelihood of obtaining the full SDD measurement result given ρ is the product of the probabilities of each projective measurement

$$L_{\text{SDD}}\left(\rho \right)=\prod_{\alpha }{\left[P_{\rho ,\alpha }\left(↑\right)\right]}^{S_{\alpha }}\;{\left[1-P_{\rho ,\alpha }\left(↑\right)\right]}^{N-S_{\alpha }}$$ (3)

with $S_{\alpha }$ the number of times ∣↑〉 was measured for the point α. In our case, we find (the derivation can be found in Supplementary Text)

$$P_{\rho ,\alpha }\left(↑\right)=\frac{1}{2}\left\{1+\text{Re}\left[\sum_{i,j}\rho_{i,j}\xi_{j,i}\left(\alpha \right)\right]\right\}$$ (4)

where we have introduced the overlap matrix $\xi_{j,i}\left(\alpha \right)=\langle j|\hat{O}\left(\frac{\alpha }{2}\right)\hat{O}\left(\frac{\alpha }{2}\right)|i\rangle$, which describes the action of the nonlinear displacement $\hat{O}\left(\frac{\alpha }{2}\right)$ on a pair of Fock states ∣i〉, ∣j〉. $\hat{O}\left(\frac{\alpha }{2}\right)$ describes displacements performed outside the Lamb-Dicke regime and therefore depends on the parameter α through Bessel functions with an expression that can be found in Supplementary Text. Computing $\xi_{j,i}\left(\alpha \right)$ in advance avoids having to perform a full quantum simulation at each step of the optimizer, speeding up the optimization process. This computation requires knowing the strength of the nonlinear SDD and the Lamb-Dicke parameter, both of which we extract by applying the operator to the ground state and by fitting the spin-excitation probability as a function of the drive time.

We also extract information about the motional-state populations (diagonal of the density matrix) by driving a single resonant sideband of order Δn for *T* different times and by measuring the spin-excitation probability (*59*). Statistics are acquired by repeating the measurement *F* times for each time t∈T. The corresponding likelihood function is

$$L_{\text{POP}}\left(\rho \right)=\prod_{t}{\left[P_{\rho ,t}\left(↑\right)\right]}^{S_{t}}\;{\left[1-P_{\rho ,t}\left(↑\right)\right]}^{F-S_{t}}$$ (5)

with $S_{t}$ the number of times ∣↑〉 was measured at time *t*. The individual success probability is

$$P_{\rho ,t}\left(↑\right)=\frac{1}{2}\sum_{i}\rho_{i,i}\left\{1+e^{-\gamma t}\text{cos}\left[g_{0}J_{\Delta n}\left(2\eta \sqrt{n+\frac{\Delta n+1}{2}}\right)t\right]\right\}$$ (6)

where γ describes the decay of the Rabi oscillation due to various decoherence channels and $g_{0}$ is determined by the laser intensity. The overall likelihood of both measurements given ρ is simply the product of the two: $L\left(\rho \right)=L_{\text{SDD}}\left(\rho \right)\;L_{\text{POP}}\left(\rho \right)$. Figure S2 of the Supplementary Materials reports the measurements of the real part of the nonlinear characteristic function and of the sideband drives used to construct L(ρ).

To reduce degrees of freedom and to ensure that the density matrix is Hermitian and positive semidefinite with unit trace, we decompose it as $\rho ={\mathrm{DD}}^{†}/\text{tr}\left[{\mathrm{DD}}^{†}\right]$, where *D* is a complex lower triangular matrix (*87*). We did not experimentally obtain results using the SDD for the imaginary part of the nonlinear characteristic function. This imaginary part encodes no information for states that are symmetric under α→−α such as (r,l)=(0,2),(1,3), but for (r,l)=(1,2),(2,3), we are left with an undetermined π/d rotation in (x,p) phase space. To break this symmetry, it is enough to select the sign of one of the imaginary coherences, which we do by imposing $\text{Im}\left(\rho_{0d}\right)=+\sqrt{\rho_{00}}\sqrt{\rho_{\mathrm{dd}}}$ (more about this symmetry can be found in Supplementary Text). In practice, the negative log-likelihood L(ρ)=−ln[L(ρ)] shares the same solution $\rho_{0}$ as L(ρ) but is simpler to implement and numerically more stable. Therefore, the functional we minimize using the ADAM optimizer (*88*) from PyTorch is

$$L\left(T\right)=-\text{ln}\left[L_{\text{SDD}}\left(T\right)\right]-\text{ln}\left[L_{\text{POP}}\left(T\right)\right]$$ (7)

### Uncertainty estimation with bootstrapping

To quantify the uncertainty on the reconstructed density matrix, we follow the bootstrapping technique (*89*). We generate a bootstrap sample by randomly resampling with replacement the whole set of the SDD measurement (M×M×N outcomes) and of the populations measurement (T×F outcomes). For each bootstrap sample *i*, we obtain the corresponding density matrix $\rho^{i}$ following the MLE procedure described above. In total, we generate *B* = 100 bootstrap samples. For each (r,l), fig. S4 shows the mean density matrix obtained by averaging over the *B* bootstrap samples, $\rho =\sum_{i}\rho_{i}/B$, together with the corresponding covariance matrix of the density matrix. Figure 2 shows the Wigner functions of the mean density matrices. The fidelity *F* of the experimental data with respect to the simulated density matrix $\rho_{\text{sim}}$ is then the average of the fidelities calculated for each bootstrap sample, $F=\sum_{i}F^{i}/B$, with $F^{i}={\left(\text{Tr}\sqrt{\sqrt{\rho^{i}}\rho_{\text{sim}}\sqrt{\rho^{i}}}\right)}^{2}$. The uncertainty range is obtained as the SD of the bootstrap sample fidelities, $\mathrm{\sigma F}=\sqrt{\sum_{i}{\left(F^{i}-F\right)}^{2}/B}$.

### Decay of the sideband Rabi oscillations

As discussed before, we extract the motional-state populations by driving a single resonant sideband and fitting it with the model in Eq. 6. This considers an exponential decay with rate γ, independent of *n*. In other works, such as (*90*), phenomenological equations have been proposed to describe the dependence of the decay rate on the Fock number. In our case, we find that the reduced $\chi^{2}$ of the fits are compatible for constant γ, for $\gamma =\gamma_{0}n$ and for $\gamma =\gamma_{0}{\left(n+1\right)}^{0.7}$ [the model from (*90*)]; therefore, we choose the first, simplest option. From the fits we obtain $\gamma =2.2\left(1\right)\;{\text{ms}}^{-1},\;1.3\left(2\right)\;{\text{ms}}^{-1},\;0.72\left(6\right)\;{\text{ms}}^{-1},\;0.65\left(7\right)\;{\text{ms}}^{-1}$ for the two-, three-, four-, and five-folded manifolds, respectively. This dependence of γ on the experimental configuration could be explained by a combination of intensity noise from the Raman beams and oscillator frequency–dependent motional dephasing.
