# Supplementary material for: Nonlinear cooling and control of a mechanical quantum harmonic oscillator
Source: Sci Adv. 2026 Nov 11;12(46):eaee5380. doi: 10.1126/sciadv.aee5380 (PMC13754035; doi:10.1126/sciadv.aee5380)
Supplement: Supplementary file 1 [file sciadv.aee5380_sm.pdf]

Science Advances
AAAS

Supplementary Materials for

Nonlinear cooling and control of a mechanical quantum harmonic oscillator

Matteo Simoni *et al.*

Corresponding author: Matteo Simoni, masimoni@phys.ethz.ch

Sci. Adv. **12**, eaee5380 (2026)
DOI: 10.1126/sciadv.aee5380

This PDF file includes:

Supplementary Text
Figs. S1 to S4

Supplementary Text

Stabilization dynamics

We study the stabilization dynamics for $(r, l) = (1, 2)$ by varying the duration of the stabilization up to 5 ms. Figure S1(a) shows that the spin population tends towards $|\uparrow\rangle$ for long stabilization times, indicating that a dark state of the operator is reached. At various stabilization times, we reconstruct the Fock population by measuring the spin oscillation while driving a fourth-order sideband. We show this in Figure S1(b), where each color identifies one of the states in the NLRE operator manifold. In Figure S1(c) we present the projection of the Fock populations into the manifolds of states for different stabilization times. Over a short timescale of less than 150 μs the motional-state population has already accumulated around the crossing point, with the projection into the whole manifold (in black) already reaching a value compatible with 1. Over a longer timescale of a few milliseconds, the population of state $|\psi_2\rangle$ leaks into $|\psi_0\rangle$ and $|\psi_1\rangle$. After 5 ms the motional state consists of a mixture of $|\psi_0\rangle$ and $|\psi_1\rangle$ with equal weights. This leakage provides direct evidence that the stabilization emerges from the destructive interference between the raising and lowering processes taking place at every Fock state.

Derivation of $P_{\rho,\alpha}(\uparrow)$ for quantum state tomography

In the following, we derive the analytical expression for $P_{\rho,\alpha}(\uparrow)$ used for MLE. Here, $\hat{O}(\frac{\alpha}{2}\hat{X})$ is the non-linear state dependent displacement and $\hat{R}(\theta, \phi) = \cos(\theta/2)\hat{\mathbb{I}} - i\sin(\theta/2)(\cos\phi\hat{X} + \sin\phi\hat{Y})$ is a general rotation of the spin. We are free to set $\phi = 0$ such that $\hat{R}(\theta = 0) = \hat{\mathbb{I}}$ and $\hat{R}(\theta = \pi) = -i\hat{X}$. After the stabilization the spin state is $|\uparrow\rangle$, hence we write the ion's initial density matrix as $\rho = \sum_{i,j} \rho_{i,j} |i \uparrow\rangle \langle j \uparrow|$.

$$\begin{aligned}
P_{\rho,\alpha}(\uparrow) &= \sum_n \langle n \uparrow| \hat{R}(\theta)\hat{O}(\tfrac{\alpha}{2}\hat{X})\, \rho\, \hat{O}^\dagger(\tfrac{\alpha}{2}\hat{X})\hat{R}^\dagger(\theta)\; |n \uparrow\rangle \\
&= \sum_{i,j} \rho_{i,j} \langle j \uparrow| \hat{O}^\dagger(\tfrac{\alpha}{2}\hat{X})\hat{R}^\dagger(\theta)\, |\uparrow\rangle \langle\uparrow| \hat{R}(\theta)\hat{O}(\tfrac{\alpha}{2}\hat{X})\, |i \uparrow\rangle \\
&= \frac{1}{4}\sum_{i,j} \rho_{i,j} \left\langle j\middle|\left(2\mathbb{I} + e^{i\theta}\,\hat{O}^\dagger(\tfrac{\alpha}{2}\hat{X})\hat{O}^\dagger(\tfrac{\alpha}{2}\hat{X}) + e^{-i\theta}\,\hat{O}^\dagger(-\tfrac{\alpha}{2}\hat{X})\hat{O}^\dagger(-\tfrac{\alpha}{2}\hat{X})\right)\middle|i\right\rangle \\
&= \frac{1}{2}\Big(1 \;+\; \cos\theta\,\mathrm{Re}[\xi(\alpha)] + \sin\theta\,\mathrm{Im}[\xi(\alpha)]\Big).
\end{aligned} \tag{S1}$$

where with Re and Im we indicate the real and imaginary part of the non-linear characteristic function $\xi(\alpha) = \sum_{i,j} \rho_{i,j}\xi_{j,i}(\alpha)$, with the overlap matrix $\xi_{j,i}(\alpha)$ defined as $\xi_{j,i}(\alpha) = \langle j|\,\hat{O}(\frac{\alpha}{2})\hat{O}(\frac{\alpha}{2})\,|i\rangle$. In our experiment we set $\theta = 0$, from which we recover the expression for $P_{\rho,\alpha}(\uparrow)$.

Non-linear state dependent displacement and density matrix symmetry

In this section we examine the symmetry properties governing the non-linear state dependent displacement (SDD) operator $\hat{O}(\alpha\hat{X})$, and the associated overlap matrix $\xi_{j,i}(\alpha) = \langle j|\,\hat{O}(\frac{\alpha}{2})\hat{O}(\frac{\alpha}{2})\,|i\rangle$. Because the experiment operates outside the Lamb-Dicke regime, $\hat{O}$ is intrinsically non-linear in the Fock number through the coupling strengths from Eq. (1). The non-linear SDD is the time evolution of the SDD Hamiltonian, which is generated by simultaneously driving first order resonant sidebands (which we call BSB and RSB) with equal strength g, where in the following we set both motional and spin phase to 0:

$$\hat{H}_{\mathrm{SDD}} = \hat{H}_{\mathrm{BSB}} + \hat{H}_{\mathrm{RSB}} = \frac{\hbar g}{2}\hat{X}\sum_{n} J_1\left(2\eta\sqrt{n+1}\right)\left(|n+1\rangle\langle n| + |n\rangle\langle n+1|\right) \tag{S2}$$

Thus, we have $\hat{O}(\alpha\hat{X}) = \exp(i\alpha\hat{X}\sum_n J_1\left(2\eta\sqrt{n+1}\right)(|n+1\rangle\langle n| + |n\rangle\langle n+1|))$, where we have identified $\alpha = gt_{\mathrm{SDD}}/2$, with t_{SDD} being the drive time of the non-linear SDD.

We now want to show that due to the symmetry of $\xi_{i,j}(\alpha)$ under exchange of i and j, measuring $\mathrm{Re}\,[\xi(\alpha)] = \mathrm{Re}\left[\sum_{i,j}\rho_{i,j}\;\xi_{j,i}(\alpha)\right]$, the real part of the non-linear characteristic function, provides information about only part of the motional state density matrix. To do this we introduce the parity operator $\hat{\mathcal{P}} = \exp(i\pi\hat{a}^\dagger\hat{a})$ (note that $\hat{\mathcal{P}}\hat{\mathcal{P}} = \mathbb{I}$). From the expression of the non-linear SDD provided above one can prove that $\hat{O}^\dagger(\alpha) = \hat{O}(-\alpha)$ and $\hat{\mathcal{P}}\hat{O}(\alpha)\hat{\mathcal{P}} = \hat{O}(-\alpha) = \hat{O}^\dagger(\alpha)$. Using these properties we derive the following relation:

$$\begin{aligned}\xi_{i,j}(\alpha) &= \langle i|\hat{O}(\tfrac{\alpha}{2})\hat{O}(\tfrac{\alpha}{2})|j\rangle = \langle i|\hat{\mathcal{P}}\hat{\mathcal{P}}\hat{O}(\tfrac{\alpha}{2})\hat{\mathcal{P}}\hat{\mathcal{P}}\hat{O}(\tfrac{\alpha}{2})\hat{\mathcal{P}}\hat{\mathcal{P}}|j\rangle \\ &= (-1)^{-i}(-1)^{j}\langle i|\hat{\mathcal{P}}\hat{O}(\tfrac{\alpha}{2})\hat{\mathcal{P}}\hat{\mathcal{P}}\hat{O}(\tfrac{\alpha}{2})\hat{\mathcal{P}}|j\rangle = (-1)^{j-i}\langle i|\hat{O}^\dagger(\tfrac{\alpha}{2})\hat{O}^\dagger(\tfrac{\alpha}{2})|j\rangle = (-1)^{j-i}\,\xi^*_{j,i}(\alpha).\end{aligned} \tag{S3}$$

This equation implies that $\mathrm{Re}\left[\xi_{i,i+d}(\alpha)\right] = (-1)^d\mathrm{Re}\left[\xi_{i+d,i}(\alpha)\right]$ and $\mathrm{Im}\left[\xi_{i,i+d}(\alpha)\right] = (-1)^{d+1}\mathrm{Im}\left[\xi_{i+d,i}(\alpha)\right]$.

Using these relations we evaluate:

$$
\begin{aligned}
\mathrm{Re}\left[\xi(\alpha)\right] &= \mathrm{Re}\left[\sum_{i,j} \rho_{i,j}\ \xi_{j,i}\right] \\
&= 2\sum_{j,d}\left(\mathrm{Re}\left[\rho_{j+2d,j}\right]\mathrm{Re}\left[\xi_{j,j+2d}\right] - \mathrm{Im}\left[\rho_{j+2d,j}\right]\mathrm{Im}\left[\xi_{j,j+2d}\right]\right) - \sum_{i}\mathrm{Re}\left[\rho_{i,i}\right]\mathrm{Re}\left[\xi_{i,i}\right] \\
&= 2\ \mathrm{Re}\left[\sum_{j,d}\rho_{j+2d,j}\xi_{j,j+2d}\right] - \mathrm{Re}\left[\sum_{i}\rho_{i,i}\xi_{i,i}\right].
\end{aligned}
\tag{S4}
$$

Here, we see that the real part of the non-linear characteristic function $\xi(\alpha)$ only depends on the even off-diagonals of the density matrix, meaning that its measurement can only reveal information about these even coherences. Similarly, measurement of the imaginary part of $\xi(\alpha)$ only reveals information about the odd coherences of the density matrix. Indeed, one can show that:

$$
\begin{aligned}
\mathrm{Im}\left[\xi(\alpha)\right] = \mathrm{Im}\left[\sum_{i,j}\rho_{i,j}\ \xi_{j,i}\right] &= 2\sum_{j,d}\left(\mathrm{Re}\left[\rho_{j+2d+1,j}\right]\mathrm{Im}\left[\xi_{j,j+2d+1}\right] + \mathrm{Im}\left[\rho_{j+2d+1,j}\right]\mathrm{Re}\left[\xi_{j,j+2d+1}\right]\right) \\
&= 2\ \mathrm{Im}\left[\sum_{j,d}\rho_{j+2d+1,j}\ \xi_{j,j+2d+1}\right].
\end{aligned}
\tag{S5}
$$

Figure S3 illustrates how knowledge of $\mathrm{Re}\left[\xi(\alpha)\right]$ determines the steady state of the NLRE operator for $(r,l) = (1,2)$ up to a phase-space rotation of $\theta = \pi/3$. In general, a d-symmetric manifold with odd d can be identified up to a rotation of $\theta = \pi/d$ through the measurement of $\mathrm{Re}\left[\xi(\alpha)\right]$. When performing MLE it is enough to impose the sign of one of the diagonals containing undetermined coherences to be positive (negative) in order to identify the manifold with $\theta = 0$ ($\theta = \pi/d$). Practically, we select $\theta = 0$ by imposing $\mathrm{Im}(\rho_{kd,(k+1)d}) = +\sqrt{\rho_{kd,\,kd}}\sqrt{\rho_{(k+1)d,\,(k+1)d}}$ for all k.

Parity measurement

In this section we derive the revival time for the parity readout of rotation symmetric bosonic states using non-linear interaction between the quantum harmonic oscillator and the spin. This formalizes the experimental procedure shown in Figure 4.

Consider a bosonic system coupled to a spin by

$$\hat{H} = g\Big(\hat{K} \otimes |\uparrow\rangle\langle\downarrow| + \hat{K}^\dagger \otimes |\downarrow\rangle\langle\uparrow|\Big) = \sum_{k=0}^{\infty} g\,\tilde{f}(k)\,|k+l\rangle\langle k| \otimes |\uparrow\rangle\langle\downarrow| + g\,\tilde{f}^*(k)\,|k\rangle\langle k+l| \otimes |\downarrow\rangle\langle\uparrow|\,, \tag{S6}$$

where $\hat{K}$ is a bosonic operator of the form $\hat{K} := \hat{a}^{\dagger\,l} f(\hat{n})$ with $\tilde{f}(k) := \langle k+l|\,\hat{a}^{\dagger\,l} f(\hat{n})\,|k\rangle$. This Hamiltonian describes a single resonant sideband which drives Rabi oscillations between the states $|k+l,\uparrow\rangle \leftrightarrows |k,\downarrow\rangle$ with a rate $g\,\tilde{f}(k)$ that is a function of the Fock occupation k. Assuming for simplicity that $\tilde{f}$ is real, the eigenstates of the Hamiltonian are then $|k,\pm\rangle = \frac{1}{\sqrt{2}}\big[\,|k,\downarrow\rangle \pm |k+l,\uparrow\rangle\,\big]$ with eigenvalues $\pm g\,\tilde{f}(k)$.

In our case, after stabilization the system starts in the state $|\Psi(0)\rangle = |\psi,\downarrow\rangle$, where ψ indicates the motional state. We apply $\hat{H}$ for time t evolving the initial state towards

$$|\Psi(t)\rangle = \sum_{k=0}^{\infty} e^{-ig\,\tilde{f}(k)t}\ \langle k,+|\,|\psi,\downarrow\rangle\,|k,+\rangle + e^{+ig\,\tilde{f}(k)t}\ \langle k,-|\,|\psi,\downarrow\rangle\,|k,-\rangle\ . \tag{S7}$$

After this evolution, we measure the spin state. The time-dependent probability to find the ion in $|\downarrow\rangle$ is

$$P_\psi(t) = \mathrm{Tr}\big[\big(\mathbf{1}\otimes|\downarrow\rangle\langle\downarrow|\big)\,|\Psi(t)\rangle\langle\Psi(t)|\,\big] = \sum_{k=0}^{\infty} |\langle k,\downarrow|\,|\Psi(t)\rangle|^2 = \sum_{k=0}^{\infty} |\psi(k)|^2\,\cos^2\!\big[g\,\tilde{f}(k)\,t\big]\,. \tag{S8}$$

where $\psi(k) = \langle k|\,|\psi\rangle$. We are interested that after a time t^* the system has undergone a revival, i.e. the probability $P_\psi(t^*) = 1$. For this purpose, let us assume that the bosonic state occupies a limited range of Fock states, i.e. $\psi(k)$ is non zero only for $k \in [a,b]$. Moreover, we make the linearity assumption of the coupling strength, i.e. we consider $\tilde{f}(k)$ to be linear in k over $[a,b]$, $\tilde{f}(k) = s_f\,k + f_0$ (note that to obtain a linear $\tilde{f}$, one requires a non-linear function f compensating the factorial terms arising from $\hat{a}^{\dagger\,l}$). Under these conditions, the probability becomes a finite Fourier cosine series:

$$P_\psi(t) = \sum_{k=a}^{b} |\psi(k)|^2 \cos^2\!\big[g\,(s_f\,k + f_0)\,t\big] = \frac{1}{2} + \frac{1}{2}\sum_{k=a}^{b} |\psi(k)|^2 \cos\!\big[2g\,(s_f\,k + f_0)\,t\big]\,. \tag{S9}$$

For arbitrary $f_0 \in \mathbb{R}$, this series is only quasi-periodic meaning that the revivals of the signal are only approximate. To make the series periodic we must make the constants s_f and f_0 commensurable. In the experiment $f_0 = 0$, so we can find the revival time t^* from the least common multiple of the

individual periods of each cosine (i.e., $\pi/(g\,|s_f|\,k)$), which can be rewritten in terms of the greatest common divisor (gcd)

$$t^* = \frac{\pi}{g\,|s_f|\,N_{\rm tot}} \qquad \text{where} \qquad N_{\rm tot} = \gcd\big(k \mid k \in [a,b]\big)\,. \tag{S10}$$

At this point in time every Fock state $|k\rangle$ with $k \in [a,b]$ will have completed exactly $k/N_{\rm tot}$ rotations. In the worst case, when $N_{\rm tot} = 1$, which is the case for a large set of integers, the revival time corresponds simply to $\pi/(g|s_f|)$.

Let us now consider $|\psi\rangle$ to have a d–modular bosonic distribution, such as cat states. In other words, we pick $|\psi\rangle \in \{|\psi_0\rangle, |\psi_1\rangle, ..., |\psi_{d-1}\rangle\}$, with $|\psi_m\rangle = \sum_k c_k\,|m + d\,k\rangle$. The revival time is then parametrized by m and d and can be written as

$$t^*(m,d) = \frac{\pi}{g\,|s_f|\,N(m,d)} \qquad \text{where} \qquad N(m,d) = \gcd\big(m{+}d\,k \mid k \in [k_a,k_b]\big) \equiv \gcd(m{+}d\,k_a, d)\,, \tag{S11}$$

where $k_a, k_b \in \mathbb{N}$ correspond to the closest multiple of d to the Fock state $|k = a\rangle$ and $|k = b\rangle$, respectively. We used in the second expression properties of the greatest common divisors. The integer $N(m,d)$ must necessarily be a multiple of the total revival time integer $N_{\rm tot}$. To find the exact revival times t^* and $t^*(m,d)$ we must in general know the specific values of a and b.

To recap, Eq. (S11) provides the time $t^*(m,d)$ for which the state $|\psi_m\rangle$ revives, i.e. $P_m(t^*(m,d)) = 1$. However, in order to perform a parity measurement that discriminates in which parity m the system is, we require that $P_{m'}(t^*(m,d)) = 0$ for all $m' \neq m$. For example, for $d = 2$, $N(0,2) = 2\gcd\big(k_a, 1\big) = 2$ and the probability of measuring $|\downarrow\rangle$ for both parity states becomes

$$P_0\big(t^*(0,2)\big) = 1 \qquad \text{and} \qquad P_1\big(t^*(0,2)\big) = \frac{1}{2} + \frac{1}{2}\sum_{k=k_a}^{k_b} c_k \cos\big[\pi\,(1+2k)\big] = 0\,, \tag{S12}$$

therefore, choosing $t^* = \pi/(2g|s_f|)$ allows to discriminate $|\psi_0\rangle$ and $|\psi_1\rangle$.

The conditions $P_m(t^*(m,d)) = 1$ and $P_{m'}(t^*(m,d)) = 0$ cannot always be fulfilled. In our case of $d = 3$, we numerically optimize the revival time $t_{\rm rev}$ to approximately satisfy both conditions and maximally distinguish $|\psi_0\rangle$ and $|\psi_1\rangle$, obtaining $P_0(t_{\rm rev}) = 0.09$ and $P_1(t_{\rm rev}) = 0.91$.

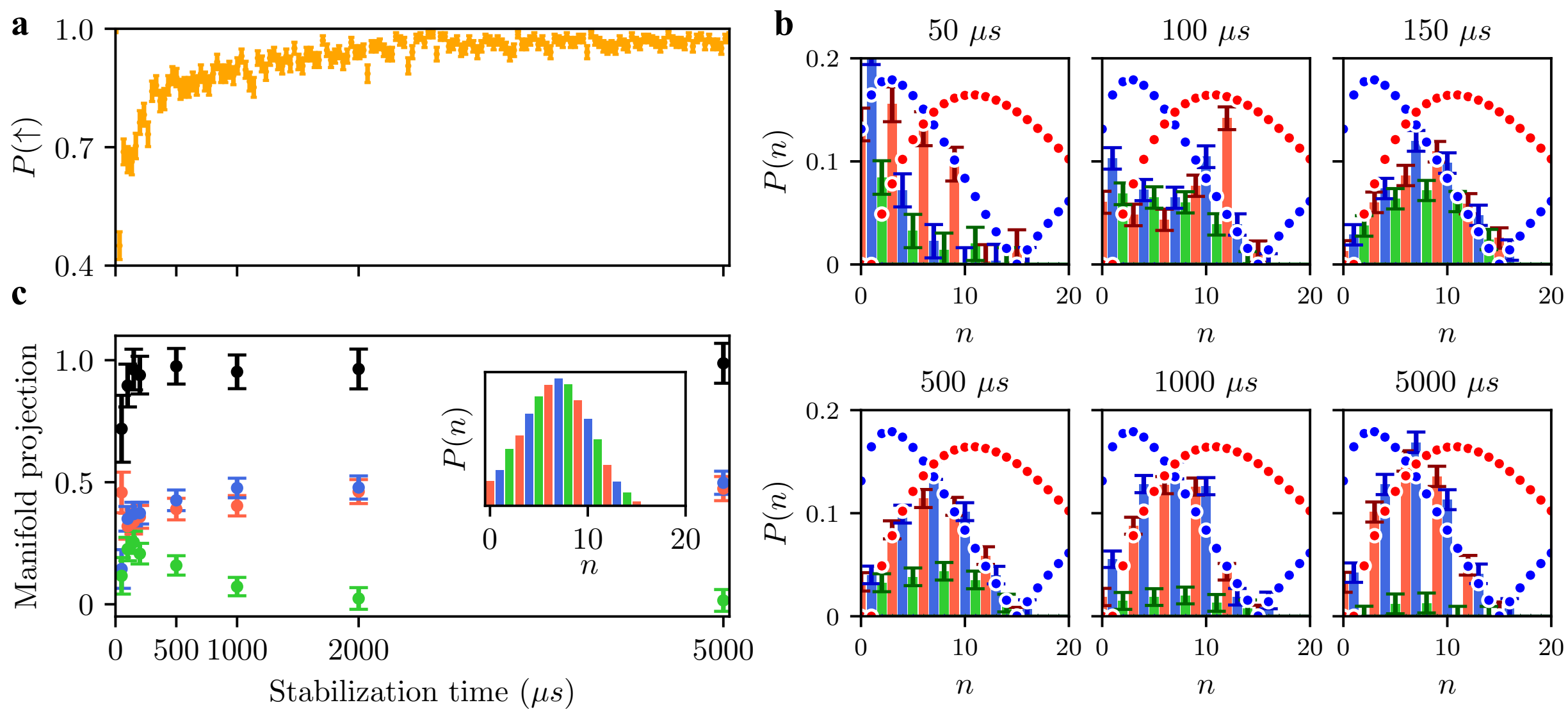

Figure S1: **NLRE stabilization dynamics**. **a.** Spin population as a function of the stabilization time. The error bars indicate the statistical uncertainty (SEM). **b.** Fock populations extracted from the experimental data using the method from (*59*) for different stabilization times from 50 μs to 5000 μs. The colors indicate the various states with $|\psi_0\rangle$ in red, $|\psi_1\rangle$ in blue and $|\psi_2\rangle$ in green. Error bars are propagated from the fit and correspond to the SEM. **c.** Projection of the reconstructed bosonic state into the $(r, l) = (1, 2)$ manifold after different stabilization times. The projection is obtained by comparing the experimental Fock populations to those of the simulated steady state (shown in the insert). In black, the projection in the manifold as a whole. Error bars on the projections are propagated from the extracted Fock distributions and correspond to the SEM.

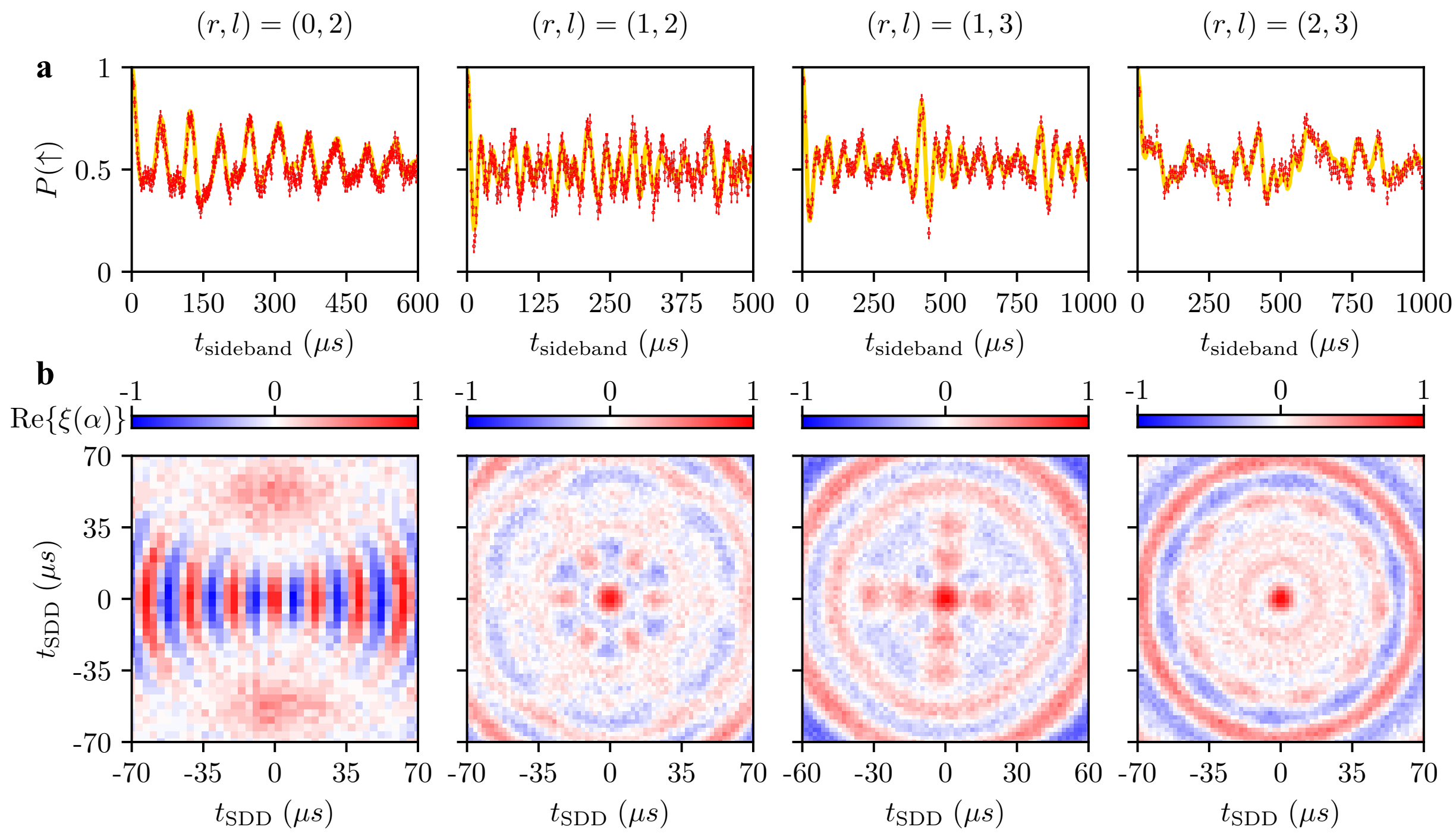

Figure S2: **NLRE sideband drives and non-linear characteristic functions**. **a.** Spin-excitation probability as a function of the sideband drive duration, used to reconstruct Fock state distributions. For $(r, l) = (0, 2), (1, 2), (1, 3), (2, 3)$ respectively the sideband order used was $\Delta n = 0, 1, 4, 4$, the number of data points is $T = 300, 250, 200, 150$ and the number of repetitions of each experimental shot is $F = 350, 150, 300, 300$. The error bars indicate the statistical uncertainty (SEM). The experimental data points are accompanied by fits that use the expression for $P_{\rho,t}(\uparrow)$ from Equation 6. **b.** Real part of the non-linear characteristic function measured by applying the non-linear SDD followed by a measurement of the spin-excitation probability as $\text{Re}\{\xi(\alpha)\} = 2P_{\rho,\alpha}(\uparrow) - 1$. For $(r, l) = (0, 2), (1, 2), (1, 3), (2, 3)$ respectively the number of data points is $M = 40, 70, 70, 70$ and the number of repetition of each experimental shot is $N = 200, 300, 300, 300$.

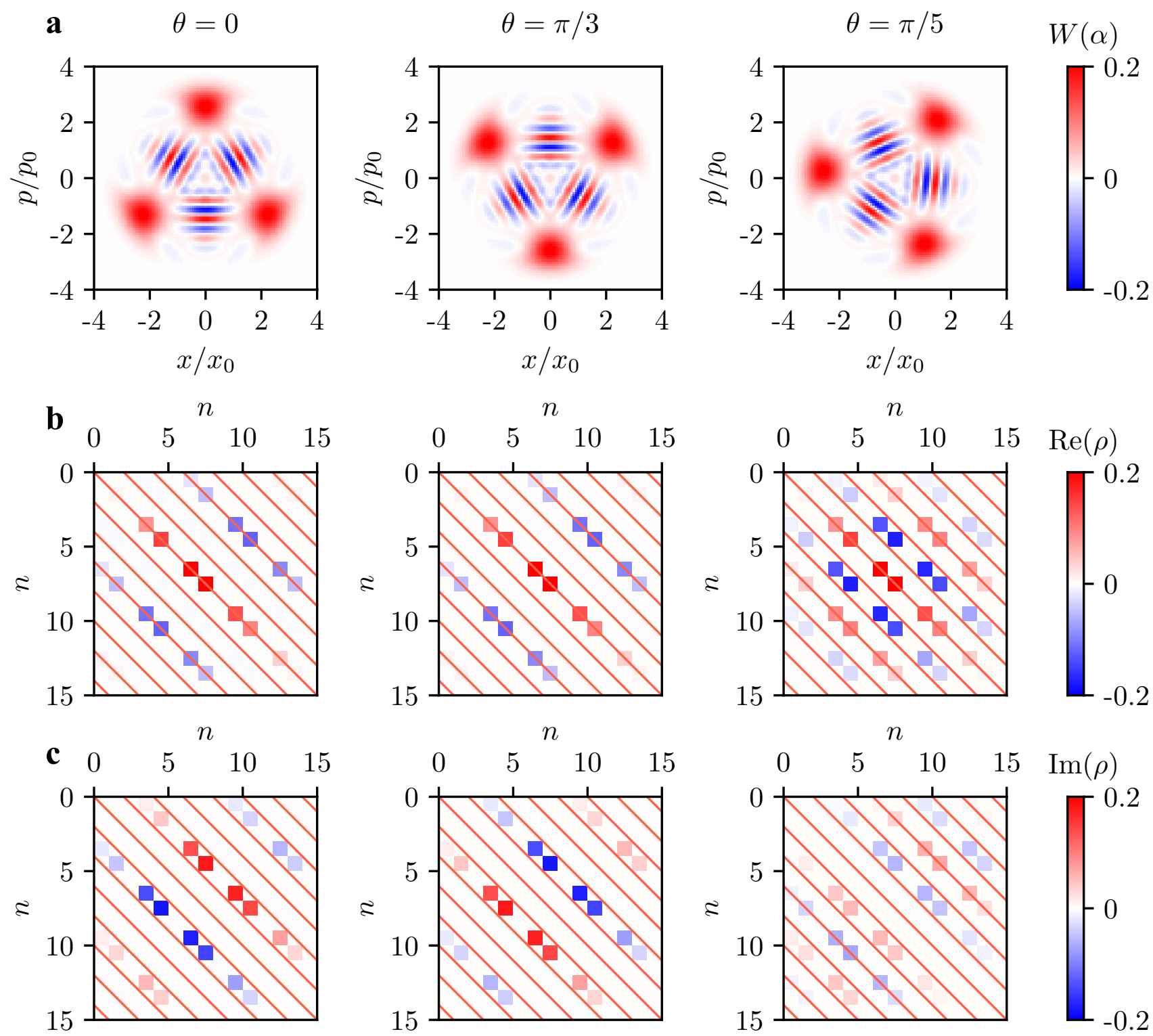

Figure S3: **Density matrix coherences and phase space rotations**. Simulated Wigner functions (**a.**), Re(ρ) (**b.**) and Im(ρ) (**c.**) of the steady state of the NLRE operator for $(r, l) = (1, 2)$. The three columns indicate a phase space rotation of $\theta = 0$, $\theta = \pi/3$ and $\theta = \pi/5$ respectively. The density matrices are accompanied by diagonal lines indicating the entries that can be obtained from the measurement of Re $[\xi(\alpha)]$, as proven in the Supplementary Text. Measuring Re $[\xi(\alpha)]$ determines ρ up to a phase space rotation of $\theta = \pi/3$ since the relevant coherences in Im(ρ) that determine this rotation do not lie on the probed diagonals. Any other rotation angle will instead involve coherences that lie in the probed diagonals and can thus be ruled out by measuring Re $[\xi(\alpha)]$. As an example, the $\theta = \pi/5$ rotation involves multiple coherences, both in Re(ρ) and in Im(ρ), that lie on the probed diagonals.

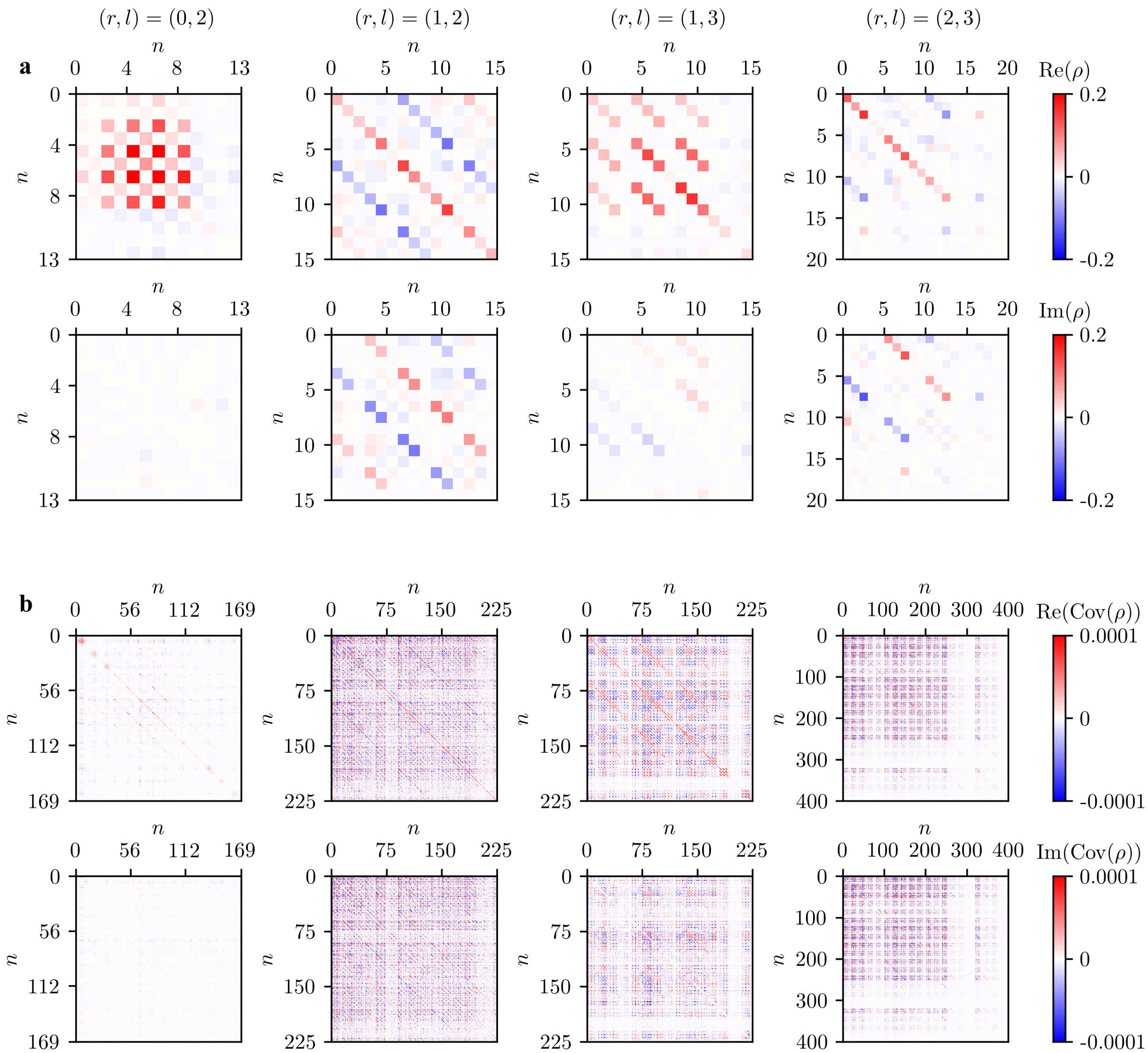

Figure S4: **Density matrices and covariance matrices from MLE**. **a.** Real and imaginary parts of the mean density matrices extracted from experimental data for $(r, l) = (0, 2), (1, 2), (1, 3), (2, 3)$. Each density matrix is the average of $B = 100$ density matrices, each obtained through MLE performed on one of the B bootstrap samples of the experimental data. **b.** Real and imaginary parts of the covariance matrices of the density matrices for $(r, l) = (0, 2), (1, 2), (1, 3), (2, 3)$.
